# Correlations of *EGFR *mutations and increases in *EGFR *and *HER2 *copy number to gefitinib response in a retrospective analysis of lung cancer patients

**DOI:** 10.1186/1471-2407-7-128

**Published:** 2007-07-13

**Authors:** Trevor J Pugh, Gwyn Bebb, Lorena Barclay, Margaret Sutcliffe, John Fee, Chris Salski, Robert O'Connor, Cheryl Ho, Nevin Murray, Barbara Melosky, John English, Jeurgen Vielkind, Doug Horsman, Janessa J Laskin, Marco A Marra

**Affiliations:** 1 Michael Smith Genome Sciences Centre, BC Cancer Agency, Vancouver, Canada; 2 Tom Baker Cancer Centre, Calgary, BC, Canada; 3 Department of Cancer Endocrinology, BC Cancer Agency, Vancouver, Canada; 4 Department of Pathology, BC Cancer Agency, Vancouver, Canada; 5 Department of Medical Oncology, BC Cancer Agency, Vancouver, Canada; 6 Department of Pathology and Laboratory Medicine, Vancouver Hospital, Vancouver, Canada

## Abstract

**Background:**

Gefitinib, a small molecule tyrosine kinase inhibitor of the Epidermal Growth Factor Receptor (*EGFR*), has shown limited efficacy in the treatment of lung cancer. Recognized clinical predictors of response to this drug, specifically female, non-smoker, Asian descent, and adenocarcinoma, together suggest a genetic basis for drug response. Recent studies have addressed the relationship between response and either sequence mutations or increased copy number of specific receptor tyrosine kinases. We set out to examine the relationship between response and the molecular status of two such kinases, *EGFR *and *HER2*, in 39 patients treated with gefitinib at the BC Cancer Agency.

**Methods:**

Archival patient material was reviewed by a pathologist and malignant cells were selectively isolated by laser microdissection or manual recovery of cells from microscope slides. Genomic DNA was extracted from 37 such patient samples and exons 18–24, coding for the tyrosine kinase domain of *EGFR*, were amplified by PCR and sequenced. *EGFR *and *HER2 *copy number status were also assessed using FISH in 26 samples. Correlations between molecular features and drug response were assessed using the two-sided Fisher's exact test.

**Results:**

Mutations previously correlated with response were detected in five tumours, four with exon 19 deletions and one with an exon 21 missense L858R point mutation. Increased gene copy number was observed in thirteen tumours, seven with *EGFR *amplification, three with *HER2 *amplification, and three with amplification of both genes. In our study cohort, a correlation was not observed between response and *EGFR *mutations (exon 19 deletion p = 0.0889, we observed a single exon 21 mutation in a non-responder) or increases in *EGFR *or *HER2 *copy number (p = 0.552 and 0.437, respectively).

**Conclusion:**

Neither mutation of *EGFR *nor increased copy number of *EGFR *or *HER2 *was diagnostic of response to gefitinib in this cohort. However, validation of these features in a larger sample set is appropriate. Identification of additional predictive biomarkers beyond *EGFR *status may be necessary to accurately predict treatment outcome.

## Background

Lung cancer is the leading cause of cancer-related death in North America with 85% of patients eventually succumbing to the disease [1]. The five year survival rate for this cancer is low (16%) compared to other cancers [1] and there exists a major need for additional therapeutic strategies in its treatment. *EGFR *has been identified as a potential therapeutic target as overexpression is observed in 40–80% late stage lung tumours and can confer a malignant phenotype in cultured cells [2]. The FDA has approved the use of two *EGFR*-targeted molecules, gefitinib ("Iressa" from Astra Zeneca) and erlotinib ("Tarceva" from Genentech/Roche) in the second- and third-line treatment of lung cancer. Both of these drugs were designed to reversibly bind the ATP-binding pocket of the *EGFR *tyrosine-kinase domain, thereby inhibiting autophosphorylation and stimulation of downstream signalling pathways resulting in inhibition of proliferation, delayed cell cycle progression, and increased apoptosis. In international phase II trials, ~28% of Japanese patients responded to gefitinib versus ~10% of patients of European descent as assessed by symptom improvement and tumour shrinkage [3,4]. These population-specific findings have suggested that response to these drugs has a genetic component although regional environmental factors have not been discounted.

Somatic mutations in the *EGFR *tyrosine-kinase domain have been correlated with reduced tumour size as a result of treatment with gefitinib [5-9]. These mutations were commonly found in patients fitting the responsive profile observed in initial and subsequent clinical studies [3,4,10], specifically female non-smokers of Asian descent. In a review of sixteen studies, *EGFR *mutations clustering around the tyrosine kinase domain ATP-binding pocket have been observed in 151 of 191 gefitinib responders (79.1%) and 11 of 19 erlotinib responders (57.9%) [11]. Confounding the model of mutation-mediated drug response is the finding that 40 of 191 gefitinib responders (20.9%) and 8 of 19 erlotinib responders (42.1%) lack *EGFR *mutations [11]. Conversely, *EGFR *mutations were seen in 40 of 355 gefitinib non-responders (11.3%) and 16 of 117 erlotinib non-responders (13.7%) [11] suggesting that somatic *EGFR *mutations are neither necessary nor sufficient for response. This suggestion is supported by the findings of a prospective trial of gefitinib in which 4 of 16 patients selected for tumours with *EGFR *mutations didn't respond to gefitinib [12]. An increasing number of studies examining the tumours of patients treated with gefitinib and erlotinib have correlated increased *EGFR *gene copy number with response [9,13,14]. Data analysis from a recent phase III trial of erlotinib has supported these observations [15]. In this trial, the response rate among patients with tumours with amplification of *EGFR *was significantly higher than those without this characteristic (20% vs. 2%) [15]. Multivariate analysis revealed that only *EGFR *expression and increased copy number were associated with erlotinib response and no correlation between base-pair mutation and response was found [15]. Increased *HER2/Neu *gene copy number has also been associated with response, particularly in the presence of increased *EGFR *copy number, *EGFR *overexpression or *EGFR *mutation [14]. Other studies have shown that tumours co-expressing *HER2 *and *EGFR *have a poor prognosis [16,17] suggesting that there is a relationship between these genes that drives pathogenesis and which may be targeted by gefitinib. Additional data are needed to explore the ability of these molecular features to predict response to *EGFR*-targeted tyrosine-kinase inhibitors [18,19].

Recently we confirmed that Asian ethnicity still predicts for response to gefitinib in a Canadian setting. We retrospectively analysed the experience of the Vancouver Cancer Centre in using gefitinib in a population in which 38% of patients are of Asian descent [10]. To test previous correlations of molecular features with response, we retrospectively analyzed diagnostic samples from this cohort of patients for somatic *EGFR *mutations as well as copy number alterations in *HER2 *and *EGFR*.

## Methods

### Patient population & assessment response

Samples for molecular analysis were drawn from patients who received gefitinib through the Extended Access Program at the BC Cancer Agency as reported by Ho et al [10] with ethics approval from the BC Cancer Agency Ethics Review Board. The criteria for enrolment in the program were the presence of histologically or cytologically confirmed locally advanced or metastatic NSCLC having received prior standard systemic or radiation therapy or being ineligible for standard treatment. Patients received gefitinib following standard systemic or radiation therapy and response was assessed radiographically according to the SWOG modification of the WHO criteria [20]. In brief, complete response (CR) was defined as a complete disappearance of disease, partial response (PR) was defined as a decrease of > 50% of the sum of the products of the maximal perpendicular dimensions of measurable lesions, stable disease (SD) was defined as the presence of no new lesions or progression of current lesions, progressive disease (PD) was defined as an increase of > 50% of the sum of the products of the maximal perpendicular dimensions of measurable lesions, the development of new lesions, recurrence of lesions that had previously disappeared or failure to return for evaluation because of symptomatic deterioration.

### Laser microdissection and DNA extraction

**Figure 1:**
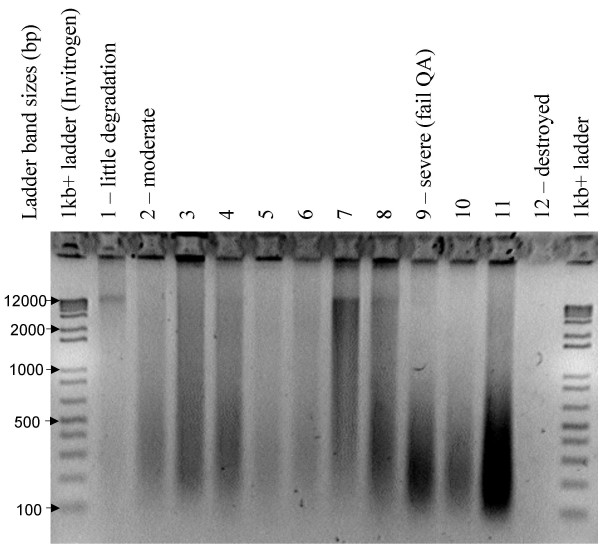
**DNA of varying quality from formalin-fixed paraffin-embedded tissues**. DNA extracted from tissue blocks is often degraded and chemically modified to varying degrees due to differences in fixation method and time, storage conditions, and nature of the tissue. Diagnostic treatments such as fixation with Bouin's (samples 9–11) or acid decalcification (sample 12) can result in severely degraded template unusable for PCR. Little (sample 1) or moderately (sample 2–8) degraded templates can be used for PCR although additional input DNA may be necessary for robust PCR. To ensure that blocks with degraded DNA were not used in labour-intensive microdissection, DNA from whole sections was extracted and qualified on a 2% agarose gel prior to microdissection of additional sections. Blocks yielding highly degraded DNA were not used in this study.

To identify tumour cell populations for laser microdissection (LM) or manual scrape, malignant cells (cytology specimens) or tissues (paraffin embedded biopsies) were reviewed by a single reference pathologist. Because the DNA extracted from formalin-fixed, paraffin-embedded tissue blocks is of variable quality, the DNA from these sources was characterized prior to microdissection. DNA was extracted from a full 8 micron section of each block using the "Laser-Microdissected Tissues" protocol of the QIAamp spin-column kit (QIAgen, Valencia, CA). The digestion volumes were increased five-fold and three final 30 uL elutions of TE (10:0.1) were performed. The DNA was quantified by PicoGreen assay (Invitrogen, Carlsbad, CA) and observed on a 2% agarose gel stained with ethidium bromide. For a block to qualify for LM, the presence of DNA fragments > 2000 bp was required (Figure 1). 40 archival samples from 37 patients were suitable for LM and yielded enough DNA of sufficient quality for PCR and sequencing.

**Figure 2:**
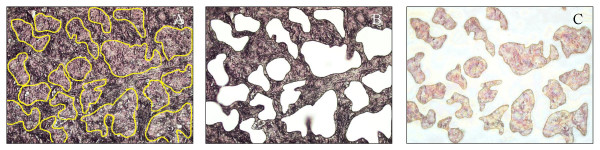
**Laser microdissection of mixed tumour & normal cell populations**. Tumour cells were microdissected using an MMI SLμCUT UV laser microdissection instrument to isolate tumour cells from surrounding normal tissue. A) Uncut lymph node tissue with metastatic tumour populations outlined in yellow. B) Normal stromal cells remaining after excision of tumour. C) Tumor cells isolated on adhesive cap.

Laser microdissection of pathologist-identified cells was performed on serial sections of paraffin blocks using either the Arcturus PixCell infra-red laser-capture or MMI SLμCUT UV laser microdissection instruments. Dissected cells were isolated onto the adhesive caps of 1.0 mL microcentrifuge tubes (Arcturus) (Figure 2). Material from cytology slides was scraped with a razor blade directly into microcentrifuge tubes and DNA extracted as described above.

### PCR and sequencing of *EGFR *exons 18–24

Exons 18–24, coding for the tyrosine kinase domain of *EGFR*, were amplified by PCR and sequenced. PCR primers were designed using human genome reference sequence acquired from the UCSC Genome Browser [21,22] (hg17_refGene_NM_005228). Primers were designed to anneal within introns at least 40 bp away from exon splice sites using the Primer3 program [23]. Sequencing tags were added to all PCR primers for downstream sequencing and experimentally optimized for annealing temperature. The DNA sequence and annealing temperatures (T_ann_) of all seven *EGFR *primer pairs are listed in Table 1. PCR reactions were performed in 20 uL and consisted of: 2.0 uL 10× Pfx Amplification Buffer (Invitrogen), 0.4 uL 50 mM MgSO_4 _(Invitrogen), 0.4 uL 10 mM dNTPs (from 100 mM stock, Invitrogen), 1 uL each of 10 uM forward and reverse primers (Invitrogen), 2.0 uL 10× PCR_x _Enchancer (Invitrogen), 0.1 uL 2.5 U/uL Pfx Polymerase (Invitrogen) with 5–10 ng template and distilled water added up to the final volume. Reactions were cycled on an MJResearch Tetrad at 95 C for 5 minutes followed by 35 cycles of 95 C for 30s T_ann _for 15s (Table 1), and 70 C for 2 minutes. PCR products were purified using the Ampure magnetic-bead-based PCR product purification system (Agencourt, Beverly, MA).

**Table 1:** PCR primers for 7 exons of the *EGFR *tyrosine kinase domain

**Exon**	**Annealing Temperature, °C (T_ann_)**	**Forward Primer Sequence**	**Reverse Primer Sequence**	**Product Length (with seq tags)**
18	60	gtgtcctggcacccaagc	ccccaccagaccatgaga	340
19	60	cagcatgtggcaccatctc	cagagcagctgccagacat	273
20	60	cattcatgcgtcttcacctg	catatccccatggcaaactc	412
21	60	agccataagtcctcgacgtg	acccagaatgtctggagagc	372
22	56	tccagagtgagttaactttttcca	ttgcatgtcagaggatataatgtaa	277
23	60	gaagcaaattgcccaagact	atttctccagggatgcaaag	413
24	56	gcaatgccatctttatcatttc	gctggcatgtgacagaacac	281

PCR primers were designed at least 40 bp from *EGFR *exons coding for the tyrosine kinase domain. Sequencing tags were added to each primer to allow sequencing of the PCR products. All forward primer sequences were prefixed with a -21M13 sequencing tag, TGTAAAACGACGGCCAGT. All reverse primer sequences were prefixed with an M13R sequencing tag, CAGGAAACAGCTATGAC. -21M13 and M13R sequencing primers were then used in the corresponding sequencing reaction to generate sequences from both strands of the PCR products.

Sequencing of PCR products was performed with standard chemistries in use by the production sequencing team at the BC Cancer Agency Michael Smith Genome Sciences Centre. Briefly, "forward" and "reverse" 1/24× reactions contained 0.33 uL BigDye Ready Reaction Mix v3.1 (ABI), 0.4 uL 15× Big Dye Buffer (50% by volume Big Dye v3.1 Sequencing Buffer (ABI), 50% by volume Tris-EDTA), 0.02 uL distilled water, and 2 uL of purified PCR product. Reactions were cycled 50 times with annealing temperatures (T_ann_) appropriate to the forward and reverse sequencing primers in use (96°C for 10s, T_ann _for 5s, 60°C for 3 mins). All reactions were precipitated in a final concentration of 70% ethanol and 10 mM EDTA and spun at 2750 g for 30 minutes to pellet sequencing products. The pellet was washed with 30 uL of 70% ethanol and air dried before resuspension in 10 uL distilled water. Sequencing reaction products were analyzed on automated ABI 3730XL sequencers and traces analyzed using the Mutation Surveyor software package (SoftGenetics, State College, PA) and the Phred/Phrap/Consed suite [24,25]. All sequences were compared against human genome sequence (NCBI accession NM_005228.3) to identify mutations and polymorphisms. Observed known polymorphisms recorded in the Single Nucleotide Polymorphism database (dbSNP) [26,27] were identified by appropriate 'rs' number. To further validate results, PCR and sequencing reactions were repeated for all samples in which an apparent mutation was observed. Correlations between clinical features and *EGFR *mutations were assessed using the two-sided Fisher's exact test.

### Copy number analysis of *EGFR *and *HER2*

To assess *EGFR *and *HER2 *copy number, fluorescent *in-situ *hybridization (FISH) was conducted using Pathvysion *EGFR *and HER-2 DNA Probe kits (Vysis, Downers Grove, IL). Formalin-fixed paraffin-embedded tissues were prepared in serial 6 um sections on positively charged Colorfrost/Plus microscope slides (Fisher Scientific, Hampton, NH). One section was H&E stained and tumour populations identified by a pathologist. Hybridization areas were marked with a diamond-tipped pencil on the back of each slide. Sections were incubated overnight at 56°C, dewaxed by exposure to xylene for 10 minutes, dehydrated in 100% ethanol for 5 minutes, and air-dried 2–4 minutes on a slide warmer set to 37–45°C. The slides were immersed in 0.2N HCl for 20 minutes, rinsed in H_2_O for 10 minutes, and incubated in 1 M NaSCN pre-treatment solution (Vysis) for 30 minutes at 80°C. After rinsing with room temperature H_2_O for 3 minutes, sections were digested with pepsin (0.25 mg/mL in 0.01N HCl) for 15–18 minutes at 37°C, and rinsed with room temperature H_2_O for 5 minutes. Tissue morphology was assessed by phase contrast microscopy to ensure sufficient digestion of the collagen matrix. Slides were dehydrated with two 4-minute treatments of 100% ethanol and air-dried 2–4 minutes on a slide warmer set to 37–45°C. 2.5-3 uL of the *EGFR*/CEP7 or *HER2*/CEP17 probe mixture was applied to the hybridization area marked on the slide and covered with a glass coverslip. Edges were sealed with rubber cement. The slides were incubated at 73°C for 5 minutes then 37°C overnight to first co-denaturate the probe and chromosomal DNA and then allow hybridization. Rubber cemented coverslips were then removed and the slides were placed in a post-hybridization wash solution (2× SSC, 0.3% NP-40) at 72°C for 2 minutes. After rinsing the slides in 1× PBS, they were air-dried in the dark for 30–60 minutes. 4 uL DAPI-1 counterstain (Vysis) was applied to the hybridization area and coverslipped. FISH analysis was performed by counting the number of signals from each probe in forty tumour nuclei on each slide.

Two approaches were used to interpret raw FISH probe counts and define gene amplification. In the first approach, the total number of *EGFR *or *HER2 *signals was divided by the total number of centromeric CEP7 or CEP17 signals and a gene/CEP ratio reported for the population of forty cells. Samples with a gene/CEP ratio ≥ 2 were defined as displaying gene amplification. The second approach applies published criteria [13] to raw FISH counts to classify patients into six strata according to the frequency of cells with specific gene copy numbers within the tumour population. The six strata, as published [13] and applied in our study, were: 1) disomy (≤2 copies in > 90% of cells); 2) low trisomy (≤2 copies in ≥40% of cells, 3 copies in 10% – 40% of the cells, ≥4 copies in < 10% of cells); 3) high trisomy (≤2 copies in ≥40% of cells, 3 copies in ≥40% of cells, ≥4 copies in < 10% of cells); 4) low polysomy (≥4 copies in 10% – 40% of cells); 5) high polysomy (≥4 copies in ≥40% of cells); and 6) gene amplification (defined by presence of tight *EGFR *gene clusters and a ratio of *EGFR *gene to chromosome of ≥2 or ≥15 copies of *EGFR *per cell in ≥10% of analyzed cells). The first approach is commonly used in practical clinical assessment of gene copy number and generally reflects the average copy number of the cell population examined. The second approach attempts to capture the degree to which gene amplification defines a cell population. While the biological significance of the second method is unknown, it has been shown to predict response in the case of gefitinib and increased *EGFR *copy number [13].

## Results

### Patient population

We previously documented the clinical characteristics of a population of 61 patients treated with gefitinib at the BC Cancer Agency between April 2002 and May 2004 [10]. In the previous study of 61 patients, those with Asian ethnicity and adenocarcinoma histology displayed a preferential response to gefitinib. Diagnostic samples from 39 of these individuals were suitable for microdissection and yielded DNA of sufficient quality for PCR and sequencing and/or copy number analysis by FISH. Microdissected materials were used to avoid masking of cancer-specific features by contaminating normal material. Figure 2 demonstrates the heterogeneous nature of a metastatic tumour and the ability of laser microdissection to separate tumour cells from surrounding normal tissue. The patient subset consisted of 23 females (59%), 17 patients of Asian descent (44%), 12 non-smokers (31%), 34 tumours of adenocarcinoma subtype (87%), and a distribution between partial response/stable disease/progressive disease of 6/14/17 (15%/33%/44%) and 2 patients lacking a response assessment. The clinical characteristics and molecular status of these patients are described in Supplemental Table 1  (see Additional file 1).

### *EGFR *tyrosine-kinase domain mutations

**Figure 3:**
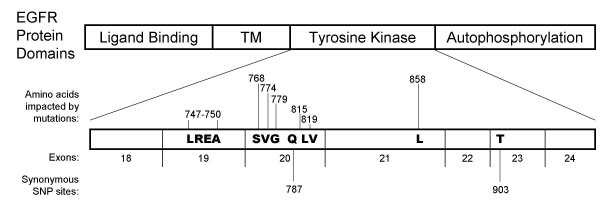
***EGFR *variant detection summary**. The seven exons coding for the tyrosine kinase domain of *EGFR *were sequenced in 37 tumours. Eight of these samples contained mutations, four with in-frame exon 19 deletions impacting L747-A750, four with a variety of exon 20 point mutations, and one with an exon 21 point mutation, L858R. Two previously documented synonymous polymorphisms were detected in this study, G2607A in exon 20 (rs10251977) and T2955C in exon 23 (rs17290643). Amino acid numbering is from the initial methionine residue of the *EGFR *protein isoform a (NCBI accession NP_005219).

We studied the DNA sequence of the *EGFR *tyrosine kinase domain in our patient samples as this domain was previously associated with increased gefitinib sensitivity [5-7]. In eight of thirty-eight tumours assessed we found ten mutations, five of which have been previously correlated with response (Figure 3). Four of these mutations were in-frame deletions or substitutions within exon 19, all of which impacted L747-A750 (Table 2) and retained the ATP-binding lysine moiety. Two of these patients were responsive to gefitinib, three were female, three were non-smokers (the smoking status for the remaining patient was unknown) and all four were of Asian descent. We resequenced the normal tissue remaining after microdissection in two of these samples and found no mutations suggesting that these mutations were specific to tumour, consistent with previous reports. The fifth mutation was a homozygous missense point mutation within exon 21 resulting in an L858R substitution (Table 3). This patient was a female non-smoker of Asian descent that did not respond to gefitinib. Three missense and two synonymous point mutations were detected in exon 20, four of which have been previously observed (Table 3). One of these mutations was in a tumour from one of the drug responsive patients that also had an exon 19 deletion. The exon 20 T790M mutation previously documented to confer resistance to gefitinib [28] was not observed. The exon 20 point mutations we observed are unlikely to be artifactual as previously postulated [29], as similar mutations were not observed in any of the other amplicons we sequenced.

**Table 2:** *EGFR *exon 19 deletions/substitution

**#**	**Sex**	**Ethnicity**	**Smoking Status**	**Source Tissue**	**Response^1^**	**I**			**K**			**E**			**L**			**R**			**E**			**A**			**T**			**S**			**P**			**K**	**a.a**.
						
						**T**	**C**	**A**	**A**	**G**	**G**	**A**	**A**	**T**	**T**	**A**	**A**	**G**	**A**	**G**	**A**	**A**	**G**	**C**	**A**	**A**	**C**	**A**	**T**	**C**	**T**	**C**	**C**	**G**	**A**	**A**	**CDS**
11	F	Asian	Unk.	Lung	PR	T	C	A	A	-	-	-	-	-	-	-	-	-	-	-	-	-	-	-	A	A	C	A	T	C	T	C	C	G	A	A	Het*
22	M	Asian	N	Lymph Node	PD	T	C	A	A	G	G	A	A	-	-	-	-	-	**C**	-	-	-	-	-	-	-	C	A	T	C	T	C	C	G	A	A	Het
25	F	Asian	N	Lung	PD	T	C	A	A	G	G	A	A	-	-	-	-	-	-	-	-	-	-	-	-	-	-	-	T	C	T	C	C	G	A	A	Del
66	F	Asian	N	Lung	PR	T	C	A	A	-	-	-	-	-	-	-	-	-	-	-	-	-	-	-	A	A	C	A	T	C	T	C	C	G	A	A	Het*

Deletions of L747-A750 were detected in *EGFR *exon 19. All samples were classified as adenocarcinoma based on histology. Deleted bases are indicated by "-". In the case of patient #22, thirteen deleted bases were replaced by a single 'C' thereby retaining the reading frame. In all cases, the ATP-binding residue K745 was retained. In the case of patients #11 and #66, a synonymous codon change results from the deletion (AAG > AAA) and the K745 ATP-binding residue is unchanged.

* = no mutations detected in normal tissue remaining after microdissection.

1 = response as measured radiographically and defined by SWOG modification of the WHO criteria.

PD = progressive disease, SD = stable disease, PR = partial response, Unk. = Unknown

**Table 3:** *EGFR *point mutations

**#**	**Sex**	**Ethnicity**	**Smoking Status**	**Source Tissue**	**Response^1^**	**Exon**	**CDS Mutation**	**Amino Acid**	**Previously Documented**
44	F	Asian	N	Pleura	PR	20	G2549 > TT C2691 > CT	S768I L815L	[32–35] none
11	F	Asian	Unk.	Lung	PR	20	G2566 > TT*	V774L	V774M [35, 36]
28	M	Caucasian	Y	Brain	SD	20	G2581 > AG	G779S	G779F [36]
35	F	Caucasian	Unk.	Brain	SD	20	G2703 > GA	V819V	[37]
47	F	Asian	N	Lung	SD	21	T2573 > GG	L858R	[5-7, 13, 15, 35, 38, 39]

Point mutations detected in *EGFR *exons 20 and 21. All samples were classified as adenocarcinoma based on histology. Point mutations altering V774 and G779 have been previously documented to result in amino acid substitutions different than those found in this study.

* = no mutations detected in normal tissue remaining after microdissection

1 = response as measured radiographically and defined by SWOG modification of the WHO criteria [20].

PD = progressive disease, SD = stable disease, PR = partial response, Unk. = Unknown

We were unable to validate the previously reported relationships between response and the presence of exon 19 mutations (p = 0.0889) or exon 21 mutations (we observed a single mutation in a non-responder). If patients exhibiting stable disease were counted among the responders ("disease control"), correlation between exon 19 deletions and response was not observed (p = 1.00). The presence of exon 19 mutations was correlated with Asian ethnicity (p = 0.0207) and non-smoking status (p = 0.0406) but not with female gender (p = 0.633) or adenocarcinoma histology (p = 1.00). When taken as a group, there were no correlations with response and exon 20 mutations (p = 0.0889), female gender (p = 0.633), non-smoking status (p = 1.00), Asian ethnicity (p = 1.00), adenocarcinoma subtype (p = 1.00), or disease control (p = 0.104).

### *EGFR *Tyrosine-kinase Domain Polymorphisms

We detected two previously documented single nucleotide polymorphisms (dbSNP rs10251977, rs17290643). Exon 20 harbours the synonymous G/A SNP rs10251977 while exon 23 contains the synonymous SNP T/C rs17290643. There was no correlation between these alleles and gefitinib response in our population.

### *EGFR & HER2 *copy number analysis

Gene copy number was assessed in our patient tumour samples as previous studies have shown a correlation between copy number increases in *EGFR *[9,13,15] or *HER2 *[14] and gefitinib response. Two techniques were used to interpret the FISH data for this analysis (Methods).

**Figure 4:**
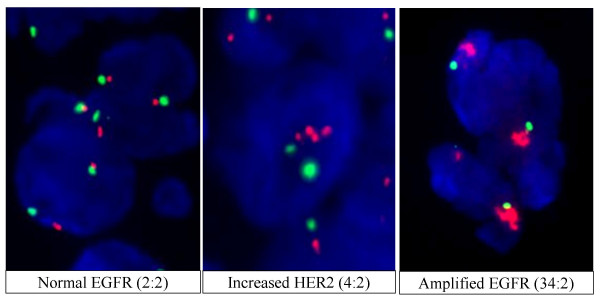
**Examples of tumours with increased gene copy number detected by FISH**. Gene copy number visualized by fluorescent in situ hybridization (FISH). Blue DAPI stain identifies the DNA present in each cell's nucleus. Red Cy5-labelled probes hybridize to the gene region targeted by each assay (*EGFR *or *HER2*). Green Cy3-labelled probes target the centromere of the chromosome appropriate for the gene-specific assay (chromosome 7 for *EGFR*, chromosome 17 for *HER2*). The ratio reported is the number of red probes/green probes (genes/chromosome) based on an average of 40 cells. A) Tumour cell without increased *EGFR *copy number, B) Tumour cell with increased *HER2 *copy number C) Tumour cell with greatly amplified *EGFR*.

Increases in *EGFR *copy number, defined as an *EGFR*/CEP7 ratio ≥ 2.0, were observed in ten of twenty-six tumours (Table 4). Of these ten, three also displayed increased *HER2 *copy number (*HER2*/CEP17 ratio ≥ 2.0). *HER2 *amplification in the absence of *EGFR *amplification was seen in three additional tumours. Examples of the varying degrees of amplification of these genes are shown in Figure 4. Increased *EGFR *copy number did not correlate with: the presence of mutation in either *EGFR *exon 19 (p = 0.130) or exon 20 (p = 1.00); increased *HER2 *copy number (p = 0.644); gender (p = 0.457); Asian ethnicity (p = 0.688); smoking status (p = 0.380); adenocarcinoma histology (p = 0.538); or response to gefitinib (p = 1.00). When patients with stable disease are counted among the responders ("disease control"), no correlation with response was observed (p = 0.210). Likewise, increased *HER2 *copy number did not correlate with: the presence of mutation of either *EGFR *exon 19 (p = 1.00) or exon 20 (p = 1.00); increased *EGFR *copy number (p = 0.644); gender (p = 0.160); Asian ethnicity (p = 0.645); smoking status (p = 0.351); adenocarcinoma histology (p = 1.00); or gefitinib response (p = 1.00) and disease control (p = 0.114).

**Table 4:** *EGFR *and *HER2 *copy number alterations

**#**	**Sex**	**Ethnicity**	**Smoking Status**	**Histology**	**Source Tissue**	**Block Type^1^**	**Response^2^**	***EGFR *Mutation**	***EGFR*/CEP7**	***HER2*/CEP17**	***EGFR *Stratification^3^**	***HER2 *Stratification^3^**
9	F	Asian	N	adeno.	Cerebellum	Tissue Block	SD	Not Sequenced	2.1	1.9	High Poly.	Low Poly.
11	F	Asian	Unk.	adeno.	Lung	Tissue Block	PR	Exon 19 Del*, Exon 20 V774L	2.7	1.5	High Poly.	High Trisomy
27	F	Caucasian	Y	SCC	Lung	Tissue Block	PD	None	2.1	1.5	High Poly.	Low Poly.
34	M	Caucasian	Y	SCC	Lung	Tissue Block	SD	None	17.3	2.6	Gene Amp.	High Poly.
36	M	Asian	Y	adeno.	Pleura	Tissue Block	SD	None	2.0	2.0	Low Poly.	Low Poly.
40	M	Caucasian	Y	adeno.	Pleura	Cell Block	SD	None	3.1	1.4	High Poly.	Low Poly.
42	M	Caucasian	Y	adeno.	Lymph Node	Tissue Block	Unk.	None	2.1	2.3	Low Poly.	High Poly.
43	F	Caucasian	Y	adeno.	Lymph Node	Tissue Block	PD	None	2.7	0.9	High Poly.	Low Trisomy
44	F	Asian	N	adeno.	Pleura	Tissue Block	PR	Exon 20 S768I, Exon 20 L815L	1.9	2.9	Low Poly.	High Poly.
56	M	Caucasian	Y	adeno.	Lung	Tissue Block	Unk.	None	1.5	2.0	High Trisomy	Low Poly.
57	M	Caucasian	Y	adeno.	Lymph Node	Tissue Block	SD	None	1.6	2.2	Low Poly.	High Poly.
64	F	Caucasian	Y									
			Pre Rx:	adeno.	Lymph Node	Tissue Block	-	None	1.0	1.2	Low Trisomy	High Trisomy
			Post Rx:	adeno.	Pericaridium	Tissue Block	SD	None	2.2	1.3	Low Poly.	Low Trisomy
66	F	Asian	N	adeno.	Lung	Tissue Block	PR	Exon 19 Del*	2.9	1.2	High Poly.	Low Trisomy

Patient data provided for samples displaying increased *EGFR *or *HER2 *copy number (Probe/CEP ratio > 2.0) or identified as FISH+ (High Polysomy or Gene Amplification)

* = no mutations detected in normal tissue remaining after microdissection

1 = source of patient material (Tissue Block = microdissected formalin-fixed paraffin-embedded tissue block; Cell Block = whole section or microdissected formalin-fixed paraffin-embedded cell block; Cytology = scraped cytology slide)

2 = response as measured radiographically and defined by SWOG modification of the WHO criteria [20].

PD = progressive disease, SD = stable disease, PR = partial response, Unk. = Unknown

3 = Copy number stratification as proposed by Cappuzzo et al [13]. (Disomy = < 2 copies in > 90% of cells, Low Trisomy = ≤2 copies in ≥40% of cells, 3 copies in 10–40% of cells, ≥4 copies in < 10% of cells, High Trisomy = ≤2 copies in ≥40% of cells, 3 copes in ≥40% of cells, ≥4 copes in < 10% of cells, Low Polysomy: ≥4 copies in 10–40% of cells, High Polysomy = ≥4 copies in 40% of cells, Gene Amplification = ≥15 copies in ≥10% of cells)

Tumours were also stratified by *EGFR *and *HER2 *copy number using the criteria proposed by Cappuzzo et al [13] (Table 4). Seven tumours were identified as FISH+ for *EGFR *amplification and four tumours were identified as FISH+ for *HER2 *amplification (high polysomy or gene amplification). Only one of these tumours was FISH+ for both *EGFR *and *HER2 *and this was the only sample to meet the *EGFR *"gene amplification" criteria as proposed by Capuzzo et al (10; ≥15 copies in ≥10% of cells). FISH+ status corresponded with an *EGFR*/CEP7 ratio ≥ 2.0 in seven of ten samples. FISH+ status corresponded with a *HER2*/CEP17 ratio ≥ 2.0 in four of six samples. There was no correlation between *EGFR *FISH+ status and mutation of either *EGFR *exon 19 (p = 0.0543) or exon 20 (p = 0.283); female gender (p = 0.378); Asian ethnicity (p = 1.00); smoking status (p = 1.00); adenocarcinoma histology (p = 0.167); response to gefitinib (p = 0.552) or disease control (p = 0.653). Likewise, *HER2 *FISH+ did not correlate with the presence of mutation of either *EGFR *exon 19 (p = 1.00) or exon 20 (p = 0.544); increased *EGFR *copy number (p = 1.00); gender (p = 0.593); Asian ethnicity (p = 0.593); smoking status (p = 1.00); adenocarcinoma histology (p = 0.408); response to gefitinib (p = 0.437) or disease control (p = 0.239).

## Discussion

In DNA sequencing studies using patient samples, contaminating normal tissue has the potential to mask tumour-specific features particularly in cases of highly heterogeneous metastatic deposits. To examine somatic features specific to tumours, we employed laser microdissection to isolate cancer cells from surrounding normal tissue. The selectivity of this technique was demonstrated by the identification of *EGFR *exon 19 deletions in the tumour populations of two patient samples but not the surrounding normal tissue remaining after microdissection.

In the evolving area of biomarkers predictive of response to *EGFR *tyrosine kinase inhibitors, two hypotheses have arisen, each claiming a specific alteration of *EGFR *is predictive of response. One hypothesis is that mutations within the *EGFR *tyrosine kinase domain targeted by these drugs are indicative of a capability to respond [5-7]. The second hypothesis is that the presence of increased gene copy number of *EGFR *or *HER2 *is a better predictor of response [13-15]. When investigating the relevance of these features to our own population of lung cancer patients treated with gefitinib, our study detected all of these features occurring both independently and coincidentally in microdissected tumour cells.

Tumours from four of thirty-eight patients contained a form of the exon 19 L747-A750 deletion and one tumour harboured the exon 21 L858R point mutation. Two of the patients with exon 19 deletions were responsive to gefitinib and were also found to have increased *EGFR *copy number. In the remaining four responders, *EGFR *mutations or gene amplifications that others previously correlated with gefitinib response [5-7] were not observed. These data are consistent with the notion that tumours reliant on amplification of a mutant *EGFR *allele may be particularly susceptible to inhibition by gefitinib. However, responders without apparent gefitinib-sensitising *EGFR *alterations may have shown characteristics of response even without treatment or may have responded due to an interaction between gefitinib and a protein other than *EGFR *[30,31]. To identify alternative genetic features mediating drug response, candidate genes influenced by receptor tyrosine kinase inhibitors need to be identified and studied in patients receiving these drugs.

In this study, we compared two methods of interpreting FISH data and defining increased gene copy number. One technique defined gene amplification as a gene/centromere (e.g. *EGFR*/CEP7) threshold ≥ 2.0 while the second technique defined "FISH+" status from the stratification of different gene/centromere ratios into varying degrees of polysomy [13]. While both of these methods identified seven tumours with *EGFR *amplification, the *EGFR*/CEP7 ratio ≥ 2 method identified an additional three tumours which were classified as "Low Polysomy" under the Cappuzzo criteria. While not originally designed for this purpose, we also applied Cappuzzo's criteria [13] to our *HER2 *FISH data. Again we saw an overlap of the samples identified by both methods as having increased *HER2 *copy number. However, as with *EGFR*, the *HER2*/CEP17 ratio method identified samples not captured by the stratification method but with ratios near the threshold of 2 for amplification. None of these patients responded to gefitinib. These results suggest a need for further refinement of criteria for defining amplification and may reflect the ability of FISH to define precise copy number. Our experience underscores the difficulty in capturing the heterogeneous nature of a tumour population with a single measurement. An understanding of the biological implications of *EGFR *gene amplification is needed to refine the predictive specificity of these tests.

## Conclusion

Recently, several studies have correlated gefitinib response with either *EGFR *mutation [5-9] or increased *EGFR *copy number [9,13-15] but the true predictive value of these features is still under debate [18,19]. While we observed *EGFR *DNA sequence mutations and increases in *EGFR *and *HER2 *gene copy number in several of our specimens, we were unable to statistically correlate the presence of any of these molecular features with response. While these findings may be due to a lack of statistical power due to our small sample size, our study differs from others in our use of a population with a large Asian component in a North American setting. Even though *EGFR *status was not a single predictive factor of drug response in our small sample set, its assessment can increase the specificity of selecting patients likely to respond to these drugs. To improve the sensitivity of screening for potential responders, additional features other than *EGFR *that mediate drug response need to be identified. Clinical criteria including gender, histology, smoking status and ethnicity, are likely to continue to play a role in the selection of patients for treatment with the *EGFR *tyrosine kinase inhibitors in NSCLC as indirect surrogates for molecular features, until completion of prospective clinical trials validating direct predictive tests of tumour biology. Such trials should be designed specifically to examine the utility of multiple molecular predictors of response concurrently.

## Competing interests

The author(s) declare that they have no competing interests.

## Authors' contributions

TJP participated in the study coordination, performed the DNA extraction, qualification, and sequence analysis, and generated drafts of the manuscript. GB conceived of the study and participated in its design as well as treated patients and identified patients for study. LB, MS, JF reviewed patient samples and performed microdissection. CS and DH carried out the FISH studies. RO served as the reference pathologist and reviewed all patient samples. CH, NM, BM treated patients and identified patients for this study. JE coordinated sample acquisition. JV oversaw the microdissection process. JL and MAM conceived of the study, participated in its design and coordination, and contributed to writing the manuscript. All authors have read and approved the final manuscript.

## Pre-publication history

The pre-publication history for this paper can be accessed here:



## Supplementary Material

Additional file 1 Supplemental Table 1 – Summary of all patient clinical data and molecular status. A table containing clinical data and molecular status of all patients studied. Click here for file
