# Supplementary material for: Correlations of EGFR mutations and increases in EGFR and HER2 copy number to gefitinib response in a retrospective analysis of lung cancer patients
Source: BMC Cancer. 2007 Jul 13;7:128. doi: 10.1186/1471-2407-7-128 (PMC1952070; doi:10.1186/1471-2407-7-128)
Supplement: Additional file 1 — Supplemental Table 1 – Summary of all patient clinical data and molecular status. A table containing clinical data and molecular status of all patients studied. [file 1471-2407-7-128-S1.doc]

**Supplemental Table 1 - Summary of all patient clinical data and molecular** status

| **#** | **Sex** | **Ethnicity** | **Smoker?** | | **Histology** | **Source**  **Tissue** | **Block Type**^1^ | **Resp-onse^2^** | ***EGFR* Mutation** | ***EGFR*/CEP7** | ***HER2*/CEP17** | ***EGFR* Stratification^3^** | ***HER2* Stratification^3^** |
| --- | --- | --- | --- | --- | --- | --- | --- | --- | --- | --- | --- | --- | --- |
| 3 | F | Caucasian | Unk. | | adeno. | Skin Nodule | Tissue Block | PD | None |  |  |  |  |
| 6 | F | Caucasian | Y | | adeno. | Lung | Tissue Block | PD | None |  |  |  |  |
| 7 | F | Caucasian | Unk. | | adeno. | Lymph Node | Tissue Block | PD | None |  |  |  |  |
| 9 | F | Asian | N | | adeno. | Cerebellum | Tissue Block | SD | Not Sequenced | 2.1 | 1.9 | High Poly. | Low Poly. |
| 10 | M | Caucasian | Unk. | | PD NSC | Lymph Node | Tissue Block | PD | None | 1.2 | 1.4 | High Trisomy | High Trisomy |
| 11 | F | Asian | Unk. | | adeno. | Lung | Tissue Block | PR | Exon 19 Del.*, Exon 20 V774L | 2.7 | 1.5 | High Poly. | High Trisomy |
| 12 | M | Asian | N | | adeno. | Lymph Node | Tissue Block | SD | None | 1.2 | 1.9 | Low Trisomy | Low Poly. |
| 14 | F | Asian | N | | adeno. | Pericardium | Tissue Block | PD | None | 1 | 1.2 | Disomy | Low Trisomy |
| 15 | M | Asian | Y | | adeno. | Lung | Tissue Block | PR | Not Sequenced | 1.1 | 1.2 | Low Trisomy | High Trisomy |
| 20 | F | Caucasian | Unk. | | adeno. | Lung | Tissue Block | PD | None | 1.1 | 1.7 | Low Trisomy | Low Poly. |
| 21 | F | Caucasian | Y | | adeno. | Lymph Node | Cytology Slide | PR | None |  |  |  |  |
| 22 | M | Asian | N | | adeno. | Lymph Node | Cytology Slide | PD | Exon 19 Del. |  |  |  |  |
| 24 | F | Caucasian | N | | adeno. | Lymph Node | Cytology Slide | SD | None |  |  |  |  |
| 25 | F | Asian | N | | adeno. | Lung | Cytology Slide | PD | Exon 19 Del. |  |  |  |  |
| 26 | F | Caucasian | Y | | adeno. | Lung | Cytology Slide | SD | None |  |  |  |  |
| 27 | F | Caucasian | Y | | SCC | Lung | Tissue Block | PD | None | 2.1 | 1.5 | High Poly. | Low Poly. |
| 28 | M | Caucasian | Y | | adeno. | Brain | Tissue Block | SD | Exon 20 G779S | 1.9 | 1.5 | Low Poly. | Low Poly. |
| 30 | M | Asian | Y | | adeno. | Brain | Tissue Block | SD | None | 1.3 | 1.2 | High Trisomy | High Trisomy |
| 33 | M | Asian | Y | | adeno. | Lung | Tissue Block | PD | None | 1.3 | 1.1 | High Trisomy | Low Trisomy |
| 34 | M | Caucasian | Y | | SCC | Lung | Tissue Block | SD | None | 17.3 | 2.6 | Gene Amp. | High Poly. |
| 35 | F | Caucasian | Unk. | | adeno. | Brain | Tissue Block | PD | Exon 20 V819V | 0.7 | 1.4 | Low Trisomy | Low Poly. |
| 36 | M | Asian | Y | | adeno. | Pleura | Tissue Block | SD | None | 2.0 | 2.0 | Low Poly. | Low Poly. |
| 37 | M | Caucasian | Y | | PD NSC | Skin Nodule | Cytology Slide | SD | None |  |  |  |  |
| 39 | M | Caucasian | Unk. | | adeno. |  | Cell Block | SD | None |  |  |  |  |
| 40 | M | Caucasian | Y | | adeno. | Pleura | Cell Block | SD | None | 3.1 | 1.4 | High Poly. | Low Poly. |
| 42 | M | Caucasian | Y | | adeno. | Lymph Node | Tissue Block | Unk. | None | 2.1 | 2.3 | Low Poly. | High Poly. |
| 43 | F | Caucasian | Y | | adeno. | Lymph Node | Tissue Block | PD | None | 2.7 | 0.9 | High Poly. | Low Trisomy |
| 44 | F | Asian | N | | adeno. | Pleura | Tissue Block | PR | Exon 20 S768I, Exon 20 L815L | 1.9 | 2.9 | Low Poly. | High Poly. |
| 47 | F | Asian | N | | adeno. | Lung | Tissue Block | PD | Exon 21 L858R | 1.2 | 1.4 | Low Trisomy | Low Poly. |
| 48 | F | Asian | N | | adeno. | Lung | Tissue Block | PD | None | 1.3 | 0.8 | High Trisomy | Disomy |
| 51 | F | Caucasian | Y | | LCC | Lymph Node | Cytology Slide | PD | None |  |  |  |  |
| 52 | F | Asian | N | | adeno. | Lymph Node | Cytology Slide | PR | None |  |  |  |  |
| 56 | M | Caucasian | Y | | adeno. | Lung | Tissue Block | Unk. | None | 1.5 | 2.0 | High Trisomy | Low Poly. |
| 57 | M | Caucasian | Y | | adeno. | Lymph Node | Tissue Block | SD | None | 1.6 | 2.2 | Low Poly. | High Poly. |
| 59 | M | Caucasian | Y | | adeno. | Skin Nodule | Tissue Block | PD | None | 1.1 | 1.4 | Low Trisomy | High Trisomy |
| 60 | F | Asian | N | | adeno. | Lymph Node | Tissue Block | SD | None | 1.1 | 1.7 | Low Trisomy | Low Poly. |
| 61 | F | Asian | Y | | adeno. | Lung | Cytology Slide | PD | None |  |  |  |  |
| 64 | F | Caucasian | Y | PreRx: | adeno. | Lymph Node | Tissue Block | - | None | 1.0 | 1.2 | Low Trisomy | High Trisomy |
|  |  |  |  | Post Rx: | adeno. | Pericaridium | Tissue Block | SD | None | 2.2 | 1.3 | Low Poly. | Low Trisomy |
| 66 | F | Asian | N | | adeno. | Lung | Tissue Block | PR | Exon 19 Del.* | 2.9 | 1.2 | High Poly. | Low Trisomy |

* = no mutations detected in normal tissue remaining after microdissection

1 = source of patient material (Tissue Block = microdissected formalin-fixed paraffin-embedded tissue block; Cell Block = whole section or microdissected formalin-fixed paraffin-embedded cell block; Cytology = scraped cytology slide)

2 = response as measured radiographically and defined by SWOG modification of the WHO criteria [20].

(PD = progressive disease, SD = stable disease, PR = partial response, Unk. = Unknown)

3 = Copy number stratification as proposed by Cappuzzo et al [13]. (Disomy = < 2 copies in > 90% of cells, Low Trisomy = ≤ 2 copies in ≥ 40% of cells, 3 copies in 10-40% of cells, ≥ 4 copies in < 10% of cells, High Trisomy = ≤ 2 copies in ≥ 40% of cells, 3 copes in ≥ 40% of cells, ≥ 4 copes in < 10% of cells, Low Polysomy: ≥ 4 copies in 10-40% of cells, High Polysomy = ≥ 4 copies in 40% of cells, Gene Amplification = ≥ 15 copies in ≥ 10% of cells)
